# A rare presentation of *Mycobacterium africanum* after two decades: a case report from Brunei Darussalam

**DOI:** 10.5365/wpsar.2022.13.3.926

**Published:** 2022-07-22

**Authors:** Abdur Rahman Rubel, Panduru Venkata Kishore, May Thu Hla Aye, Nor Azian Hafneh, Vui Heng Chong

**Affiliations:** a Department of Medicine, Pengiran Muda Mahkota Pengiran Muda Haji Al-Muhtadee Billah Hospital, Tutong, Brunei Darussalam.; b Division of Respiratory Medicine, Department of Medicine, Raja Isteri Pengiran Anak Saleha Hospital, Bandar Seri Begawan, Brunei Darussalam.; c National Mycobacteria Reference Laboratory, Department of Laboratory Services, Ministry of Health, Bandar Seri Begawan, Brunei Darussalam.; d Pengiran Anak Puteri Rashidah Sa’adatul Bolkiah Institute of Health Sciences, Universiti Brunei Darussalam, Brunei Darussalam.

## Abstract

*Mycobacterium africanum* is endemic to West Africa and is rare outside this region. Most of the people infected with  *M. africanum* outside Africa are migrants from affected parts of Africa. We report a rare case of pulmonary tuberculosis (TB) secondary to *M. africanum* in a man in Brunei Darussalam who had lived and worked in Guinea, West Africa for 6 years more than 20 years ago. He had been well until December 2020, when he presented with a chronic cough and was diagnosed with coinfections of *Klebsiella pneumoniae* and *M. africanum*, and newly diagnosed diabetes mellitus. This case highlights an interesting manifestation of pulmonary TB secondary to *M. africanum* in a patient whose last exposure was 20 years ago, contributed to by development of diabetes mellitus.

Tuberculosis (TB) remains endemic in many parts of the world and pulmonary TB (PTB) is the most common manifestation. TB is usually caused by *Mycobacterium tuberculosis*. (*1*) However, in some parts of the world, variants predominate; for example, in West and Central Africa, *M. africanum* predominates, accounting for over 50% of PTB cases. (*2*, *3*) *M. africanum* comprises two phylogenetically distinct lineages within the *M. tuberculosis* complex (MTBC): *M. africanum* West African 1 and *M. africanum* West African 2. (*2*, *3*) Cases of *M. africanum* outside Africa are rare and often occur in people who originate from affected regions. (*4*)

Cases have been reported in England, France, Germany, Spain and the United States of America (USA), (*2*) and local transmission outside endemic regions has also been reported. A study from Norway reported a cluster of six cases of *M. africanum* originating from a single imported case. (*5*) Three patients were from countries in West Africa, and the other three were from south  Asia and the Caribbean, where *M. africanum* is not known to be present. Four of the six patients had lived in Norway for more than 10  years, and the other two for 3–9 years. The six cases were diagnosed over a 3-year period (2016–2018). (*5*) Prior to this report, no cases of *M. africanum* have been reported in the Western Pacific Region.

## THE CASE

### Case identification

**Fig. 1:**
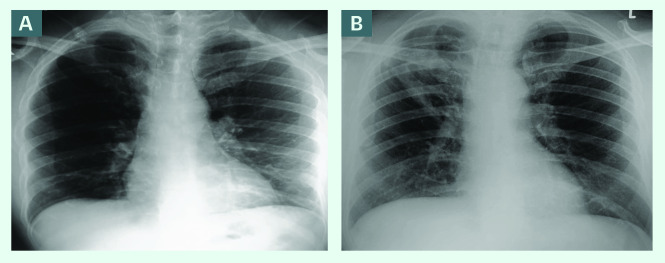
A) Chest X-ray from 2014 with normal findings, and B) from presentation in December 2020, showing fibrosis with cavitation in the upper zone on the right

A 52-year-old Malaysian man living in Brunei Darussalam who had been previously well presented with a chronic cough that had recently become productive with greenish-yellow sputum. He also reported weight loss of 3 kg in recent months. Several courses of antibiotics prescribed by a private doctor had been ineffective. A chest X-ray (CXR) showed pleural parenchymal lesions with fibrosis in the right upper zone with cavitation. His past medical history was insignificant, apart from a CXR (February 2018) done as part of occupational health screening, which showed pulmonary fibrosis in the right upper zone. CXR done before this in 2014 was normal (**Fig. 1**). He was referred for evaluation but cancelled his appointment because he was well. He is a smoker of 24 pack-years and does not consume alcohol. He reported no past history, family history or contact with anyone with TB. He was referred to the hospital for evaluation for PTB based on his history of chronic cough and CXR findings.

[**insert** Figure 1]

Clinical examination revealed minimal coarse crepitations in the right upper lung. Blood investigation showed elevated white cell count, elevated inflammatory markers, hyperlipidaemia and hyperglycaemia.

### Laboratory investigations

Three consecutive morning sputum specimens were sent to the National Mycobacteria Reference Laboratory, Department of Laboratory Services, Brunei Darussalam. In brief, the specimens were decontaminated and inoculated in both liquid (Mycobacterium growth indicator tube, Becton Dickinson, NJ, USA) and Lowenstein-Jensen slant (Becton Dickinson). Simultaneously, an aliquot of concentrated specimen was prepared for Auramine O Fluorescent Stain Kit M (Becton Dickinson). Smear-positive samples were screened for the presence of MTBC DNA and drug (rifampicin and isoniazid) resistance genes using GenoType MTBDRplus version 2.0 (Hain Lifescience GmbH, Germany), performed according to the manufacturer’s instructions. Further species differentiation within MTBC was identified using GenoType MTBC version 1 (Hain Lifescience GmbH). After reverse hybridization, the final step was a line-probe assay (LPA), which involved fixing the test strips on a designated sheet and interpretation according to the specific species or mutation band profile provided by the manufacturer.

Sputum culture isolated *Klebsiella pneumoniae* and sensitivity testing showed it to be sensitive to all antibiotics apart from ampicillin. All three morning sputum smears also came back positive for acid fast bacilli (AFB), confirming the diagnosis of PTB and *K. pneumoniae* coinfection. Serum glycosylated haemoglobin came back as 9.3% (reference: < 6.5%), confirming a new diagnosis of diabetes mellitus. AFB culture identification (LPA, GenoType MTBC) came back as *M. africanum*. HIV screening was negative.

### Treatment

The patient was started on a course of antibiotics while waiting for PTB investigations, after which he was started on anti-diabetes treatment (metformin 1000 mg twice daily and linagliptin 5 mg once daily); also, glucose control improved, with a glucose range of 5–10 mmol/L.

The patient was started on standard anti-tubercular treatment (ATT) as per World Health Organization guidelines, with 2 months of intensive treatment with isoniazid, rifampicin, pyrazinamide and ethambutol, followed by  4 months of isoniazid and rifampicin. His treatment while waiting for three consecutive negative sputum AFB tests was only complicated by mild anti-TB side-effects. On day 17 of ATT, he developed an urticarial reaction that resolved with regular antihistamines. On day 26, he developed deranged liver function tests (LFT). Hepatotoxic drugs were withheld and the patient was treated with ethambutol and second-line levofloxacin in the interim period. Once LFT normalized, ATT re-challenge with the sequential introduction of main-line ATT was achieved.

The patient completed the extended intensive-phase therapy followed by the continuation phase without any further adverse events. CXR after completion of treatment, 9 months after diagnosis, showed resolution of cavities, leaving only fibrotic changes in the right upper zone. His glycaemic control initially improved to 9.3% but later deteriorated as he had stopped his diabetes treatment due to financial issues that were compounded by the coronavirus disease (COVID-19) pandemic.

### Case history

We revisited the patient’s history, which revealed that he had lived in a small village in Guinea, West Africa from 1995 to 2000. He could not recall having contact with anyone who had chronic cough or symptoms of TB. Apart from the occasional bout of influenza, he had been well during his 6 years of living there. He then returned to his home country of Malaysia and subsequently moved to Brunei Darussalam in 2011. His family reside in Malaysia and are all well. He reported no history of contact with his former African colleagues and had not returned to Africa.

## Discussion

Humans are the only natural reservoir for *M. africanum*, which is usually transmitted by inhalation of infected droplets. However, cases of *M. africanum* infection in animals such as monkeys and cows have been reported. (*2*, *6*) A study from Bangladesh reported *M. africanum* type I identified through spoligotyping in autopsied lung tissue homogenate samples of four cows, probably infected through a farm caretaker. (*2*)

Clinical manifestations of *M. africanum* are similar to *M. tuberculosis* but have a more indolent course and less severe symptoms. (*7*) The infection may have host specificity, be influenced by factors such as age, and have less severe cough symptoms and slow progression to disease, showing the lower virulence of *M. africanum* compared with *M. tuberculosis*. A study from endemic parts of Africa has shown that patients with *M. africanum* had shorter duration of symptoms but more severe changes on chest imaging. (*7*) Those of older age and with conditions that affect the immune system (e.g. HIV and diabetes mellitus) are at higher risk. A recent study from Brunei Darussalam reported that a third of TB patients have underlying diabetes at or within 6 months of diagnosis of TB, highlighting the importance of diabetes as a risk factor in patients with TB. (*8*)

Smoking has also been shown to contribute to the risk of poor outcomes from TB. A large study of patients from 32 high TB burden countries reported an estimated 17.6% (95% confidence interval [CI]: 8.4–21.4) of TB cases and 15.2% (95% CI: 1.8–31.9) of TB mortality were attributable to smoking. (*9*) In our case, smoking is likely also a contributing factor in addition to diabetes mellitus. TB remains endemic in Brunei Darussalam, with an average of 227 cases recorded every year, for a rate of 54 per 100 000 population per year. (*8*) This case represents the first case of *M. africanum* recorded in Brunei Darussalam. In fact, literature searches failed to locate any report of *M. africanum* in South-East Asia.

This case is interesting from several aspects. First, our patient is from South-East Asia and his only exposure was during a period of residence in Guinea. There was no history of other possible exposures after returning from Guinea. Second, PTB manifested more than 20 years after exposure. In the interim period, he had been well, apart from CXR findings during an occupational health screening. He probably manifested the disease after he developed diabetes mellitus, which was diagnosed simultaneously with PTB. Apart from diabetes mellitus, there was no evidence of other conditions that can cause immune suppression. An HIV test was negative and there was no clinical evidence of underlying malignancy. Furthermore, his condition improved after starting treatment for PTB and diabetes mellitus.

TB remains an important public health problem, causing more than a million deaths each year, especially in developing countries. (*10*) With effective and timely treatment, TB is curable. Therefore, enhanced surveillance and reporting remain an integral component and should be continuously monitored and improved, especially in areas where TB remains endemic, including the Western Pacific Region. This is especially true as pandemic-related travel restrictions are eased, resulting in increasing population movement, which can lead to the appearance of TB strains in non-endemic regions.

## Conclusion

This case represents the first case of *M. africanum* recorded in Brunei Darussalam. Our patient’s only risk factor was having lived in Africa 20 years ago and this unusual manifestation probably resulted from development of underlying diabetes mellitus. Clinicians need to consider the possibility of *M. africanum* in any person with a history of travel to an endemic region, even after such a long interval. In addition, our case highlights that TB can manifest at any time, especially with the presence of underlying risk factors such as diabetes mellitus and heavy smoking.
